# High-Altitude *Plasmodium falciparum* and *Plasmodium vivax* Reservoirs in Ethiopia Are Not Linked to Recent Travel

**DOI:** 10.1093/ofid/ofag247

**Published:** 2026-04-30

**Authors:** Yalemwork Ewnetu, Tolulope Adeyemi Kayode, Colins O Oduma, Temitope M Adeyemi-Kayode, Aragaw Zemene, Wossenseged Lemma, Nega Berhane, Cristian Koepfli

**Affiliations:** Department of Medical Biotechnology, Institute of Biotechnology, University of Gondar, Gondar, Ethiopia; Gondar University Compressive Specialized Hospital, University of Gondar, Gondar, Ethiopia; Department of Biological Sciences, University of Notre Dame, Notre Dame, Indiana, USA; Department of Biochemistry and Molecular Biology, Egerton University, Nakuru, Kenya; Centre for Global Health Research, Kenya Medical Research Institute, Kisumu, Kenya; Department of Biological Sciences, University of Notre Dame, Notre Dame, Indiana, USA; Department of Medical Biotechnology, Institute of Biotechnology, University of Gondar, Gondar, Ethiopia; Department of Medical Parasitology, School of Biomedical and Laboratory Sciences, Collage of Medicine and Health Sciences, University of Gondar, Gondar, Ethiopia; Department of Medical Biotechnology, Institute of Biotechnology, University of Gondar, Gondar, Ethiopia; Department of Biological Sciences, University of Notre Dame, Notre Dame, Indiana, USA

**Keywords:** highland malaria, *hrp2* deletion, imported infection, qPCR, subclinical malaria

## Abstract

**Background:**

The Ethiopian highlands >2000 m above sea level are classified as experiencing very low malaria transmission, but clinical case numbers have increased in recent years. Transmission intensity, and how many infections are imported from the lowlands, are not known.

**Methods:**

Surveys on subclinical prevalence were conducted in 8 sites across an altitude range of <1900 to 2900 m. Samples were screened by quantitative polymerase chain reaction (qPCR) and microscopy or rapid diagnostic testing (RDT). Data on recent travel were collected from subclinical and clinical individuals.

**Result:**

Among 1241 subclinical individuals, *P. falciparum* prevalence by qPCR decreased from 67% at 1850 m to 16% at 2850 m. *P. vivax* prevalence ranged from 10% to 34%, with no clear altitude trend; 20.3% of individuals reported recent travel, which did not affect risk of infection. Among 1775 microscopy-positive clinical patients in 8 health centers, recent travel was reported by 2%–16% of patients at altitudes <2500 m, 21% in a health center at 2600 m, and 100% at 2830 m. *P. falciparum* RDT diagnosis based on LDH detected nearly twice as many subclinical infections than diagnosis based on HRP2.

**Conclusions:**

High prevalence at altitudes up to 2800 m, and a minority of individuals reporting recent travel, might indicate sustained local transmission in the Ethiopian highlands, though undocumented travel and *P. vivax* relapses limit the ability to accurately estimate the proportion of infections being imported. Sites up to 2500 m should be considered endemic for malaria, and control interventions need to be intensified.

## Graphical Abstract

**Graphical Abstract:**
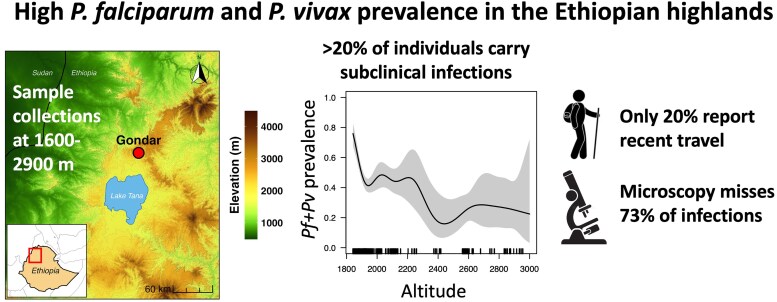

Based on the Ethiopian National Malaria Control Program (NMCP), malaria is rare at altitudes >2000 m above sea level, and absent >2500 m [1]. Clinical cases in the highlands are assumed to be imported from the lowlands. Every year, >400 000 individuals travel from the highlands to the lowlands, mostly for work in agriculture [2]. Their return after the harvest in November/December coincides with the peak of case numbers in the highlands, which are observed from September to December, following heavy rainfalls in July and August. In the hypo-endemic highland fringe zone at 1750–2000 m, malaria is also believed to be rare apart from cyclic malaria epidemics that in the past occurred every 5–8 years [1, 3].

In Ethiopia, *Plasmodium falciparum* and *Plasmodium vivax* are co-endemic, requiring tailored approaches for diagnosis, treatment, and surveillance. *P. falciparum hrp2*/*3* gene deletions are frequent in Ethiopia, adding to the complexities of malaria diagnosis. The most sensitive rapid diagnostic tests (RDTs) for *P. falciparum* detect the HRP2 and HRP3 proteins.

Wherever malaria is endemic, subclinical (asymptomatic) infections are frequent and often the source of >90% of transmission [4–7]. Understanding the prevalence of subclinical infections is a parameter to classify transmission intensity that is used by the World Health Organization (WHO) [8] and national malaria control programs [9]. Global prevalence data are compiled by the Malaria Atlas [10]. Given the low density of many subclinical infections, surveillance based on microscopy and RDTs misses many of them. Molecular methods are needed to determine the size of the subclinical reservoir. Interpretation of prevalence data requires caution, as untreated infections can persist for many months, and many *P. vivax* infections are the result of relapses and thus do not reflect recent transmission [11, 12].

A few studies and anecdotal reports have suggested that many patients presenting with clinical malaria in the Ethiopian highlands did not travel recently [13]. The origins of clinical cases observed in the Ethiopian highlands and highland fringe areas, and the intensity of local transmission, are not known. Here, we studied subclinical infection prevalence by microscopy, RDT, and quantitative polymerase chain reaction (qPCR) in 8 sites across an altitude range from 1800 to 2900 m. We estimated the proportion of the general population and of clinical patients that recently traveled to the lowlands. Further, we determined whether HRP2 or LDH-based RDTs should be used to screen for subclinical *P. falciparum* infections. We show high prevalence even at altitudes >2500. Most infected individuals did not report recent travel to the lowlands.

## METHODS

### Ethical Approval

Ethical approval was obtained from University of Gondar Vice President of Research & Community Service (approval No. VR/RTT/05/422/2021) and the University of Notre Dame Institutional Review Board (approval No. 19-08-5511). Informed written consent was collected from each individual or, in the case of minors, from the legal guardian before sample collection.

### Study Site

**Figure 1.**
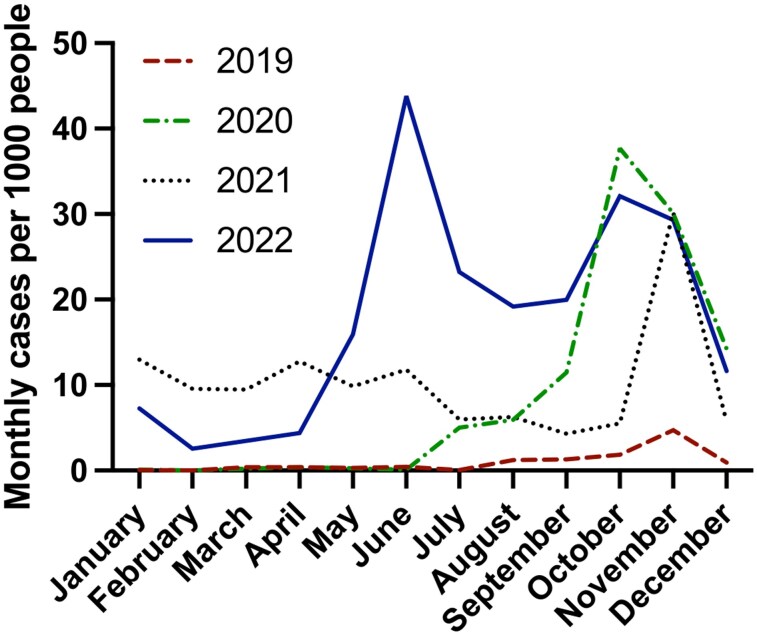
Monthly malaria incidence in Gondar Zuria District highland fringe kebeles from 2019 to 2022.

The study was conducted in Gondar Zuria District in the Central Gondar Zone in Amhara Regional state, Ethiopia. In this part of Ethiopia, malaria is seasonal, with a main peak typically occurring between November and January following heavy rains, and a minor peak in May and June. According to Gondar Zuria District Health Bureau data, there was a strong increase in annual incidence in the district, from 15.3/1000 in 2019 (3524 confirmed cases) to 158.6/1000 people (38 557 confirmed cases) in 2022, following the coronavirus disease 2019 (COVID-19) pandemic. The high number in 2022 was mostly due to an unusually high peak in case numbers in May–June (Figure 1).

Subclinical prevalence was assessed in 7 kebeles, Hamsafej, Infraz, Maksegnit, Tach Teda, Gondar City, Dabat, and Ambageorgis. In 2022, the population of these 7 kebeles was ∼50 014 and the number of confirmed malaria cases was 8866 (4666 *Pf* and 4200 *Pv*), resulting in an incidence of 177.32/1000 people (93.3 for *Pf* and 83.98 for *Pv*). In 2022, indoor residual spraying (IRS) and bed net campaigns were conducted in kebeles with an incidence of >100/1000. Among the study kebeles, Hamsafej was the only one to receive these interventions.

### Sample Collection

#### Reactive Case Detection

Between May and September 2022, during the low-transmission season, clinical cases diagnosed by microscopy in 7 health centers were followed up to their households within 7 days. Samples were collected from the household members of the index case and their neighbors (defined as households within 200 m of the index case household). Individuals were not enrolled if they reported antimalarial treatment within 3 weeks before sample collection. Initially, control households were defined as households at a distance of >1 km from the index case household with no history of malaria in the 6 months before sample collection. It was, however, virtually impossible to find households with no history of recent malaria. Further, given the unstable political situation in the region at the time of the study, it was safe to visit index patient households and their immediate neighbors, but it was deemed unsafe to visit random households. As a result, very few control household samples were collected, and those were excluded from analysis.

Demographic data, bed net use, whether the house was sprayed during an IRS campaign, and data on travel to a different woreda (district) in the 4 weeks before sample collection were recorded. Travel history was collected as a risk factor for infection and not used to definitively classify infections as imported or locally acquired.

Approximately 250 µL of blood was collected by finger prick in EDTA microtainers. RDTs were run on site, a slide prepared for microscopy, and dried blood spot (DBS) prepared for qPCR. Individuals testing positive by RDT were referred to the nearest health clinic for treatment. DBS were sent to the University of Notre Dame for qPCR. Slides were examined by 2 experienced microscopists (though they were not WHO-certified expert microscopists). *Plasmodium* infection was ruled out if no parasites were seen after examination of at least 100 microscopic fields with a 100× objective. Samples were screened with the SD BIOLINE Malaria Ag P.f./P.v. (HRP2/pLDH; LOT 05DDF012A) RDT. This RDT was used by health centers and was available locally at the time of the survey.

#### Clinical Cases

To determine the proportion of patients with confirmed malaria who recently traveled to the lowlands, individuals with microscopically confirmed *P. falciparum* and/or *P. vivax* infection were enrolled across 7 health centers, and travel in the 4 weeks prior was recorded. All individuals testing positive by local diagnosis were eligible to be enrolled.

#### Cross-Sectional Survey

To compare the sensitivity of different RDTs and targets (HRP2 vs LDH), samples were collected in 4 kebeles in Central Gondar Zone in August 2022. Households were randomly selected, and all individuals >1 year of age were invited to participate, irrespective of the presence of symptoms of febrile illness. The Rapigen Biocredit Malaria Ag Pf (pLDH/HRPII, lot No. H052BSA002) RDT was used to compare the sensitivity of HRP2 and LDH. The RDT for *P. falciparum* contains separate test bands for HRP2 and LDH. In previous studies in Burundi and Ghana, it showed high sensitivity in clinical and subclinical patients compared with previous-generation RDTs [14, 15]. The Biocredit RDT was compared with 2 RDTs that had been used by the Ethiopian NMCP, namely the Abbott Bioline Malaria Ag Pf/Pv and the AccessBio CareStart Combo Pf/Pv. These RDTs detect *P. falciparum* based on HRP2 and *P. vivax* based on LHD. Procedures for sample collection, microscopy, and sample preparation for qPCR were identical to the ones for reactive case detection (RCD).

### qPCR and *P. falciparum hrp2*/*3* Deletion Typing

DNA was extracted from the DBS using the NucleoMag Blood Kit following a published protocol [16]. Five punches, each ∼3 mm in diameter, were used for extraction; 4 µL of DNA was added to a total reaction volume of 12 µL and screened for *P. falciparum* using the multicopy *var*ATS assay, and for *P. vivax* an assay targeting the mitochondrial *cox1* gene was used [17, 18]. The limit of detection of these assays, defined as the 95% probability of detection, is 2–3 parasites/µL blood [16]. Samples positive for *P. falciparum* were typed for *hrp2*/*3* deletion by digital PCR [19].

### Data Analysis

In the absence of preliminary data on infection prevalence and the proportion of individuals with recent travel, no formal sample size calculation could be conducted. Data were analyzed using STATA 16. Mixed-effects logistic regression analysis including household and cluster (defined as all samples from index case–neighboring households) as random effects was conducted to compare prevalence across groups, including 95% CIs. McNemar tests were conducted to compare the agreement between different RDTs. In addition to calculating RDT sensitivity against all samples positive by qPCR as the gold standard, a threshold of 20 parasites/µL by qPCR was used as very low-density samples are not expected to be detected by RDT. To visualize changes in PCR positivity across altitude, a generalized additive model was calculated in R, version 4.2.3.

## RESULTS

### Prevalence of Subclinical Infection Across All Sites

Following up clinical patients, data by RDT, microscopy, and qPCR were obtained for 1241 study subjects residing in 344 households (129 index case households and 215 neighboring households). Prevalence by microscopy, RDT, and qPCR is given in Table 1. Overall, by qPCR 34.5% (428/1241) of individuals tested positive for *P. falciparum*, 21.5% (267/1241) for *P. vivax*, and 45.6% (566/1241) for the 2 species combined; 10.4% (129/1241) of individuals carried mixed-species infections of *P. falciparum* and *P. vivax*. Across both species, microscopy missed 72.3% (423/585) and RDT missed 73.3% (414/585) of qPCR-positive infections. Geometric mean parasite density by qPCR was 11.7 parasites/µL for *P. falciparum* and 6.3 parasites/µL for *P. vivax*.

**Table 1.** Prevalence of Infection by Microscopy, RDT, and qPCR

	*P. falciparum*	*P. vivax*	Combined Positive
Microscopy	10.9% (135/1241)	4.4% (55/1241)	14.2% (176/1241)
RDT	11.0% (136/1241)	2.7% (34/1241)	13.1% (162/1241)
qPCR	34.5% (428/1241)	21.5% (267/1241)	45.6% (566/1241)

Abbreviations: qPCR, quantitative polymerase chain reaction; RDT, rapid diagnostic test.

Table 2 shows the demographic composition of the study population. No significant differences in prevalence between index case and neighboring households were observed by qPCR for *P. falciparum* (*P* = .863), *P. vivax* (*P* = .246), or both species combined (*P* = .526). The prevalence of both species combined was >40% across all age groups. *P. falciparum* prevalence differed between age groups and peaked in individuals aged 5–15 years (*P* = .0095) (), while *P. vivax* prevalence was higher in children <5 years and adolescents up to 15 years compared with adults (*P* = .0093). No significant differences were observed in prevalence among sexes for *P. falciparum* (*P* = .065), *P. vivax* (*P* = .242), or both species combined (*P* = .095).

**Table 2.** Univariate Predictors of Infection by qPCR

Age (n = 1237)	No.	*P. falciparum* [95% CI], %	*P*	*P. vivax* [95% CI], %	*P*	Combined Positive [95% CI], %	*P*
<5	121	29.8 [22.4–38.4]		26.4 [17.5–37.9]	…	45.5 [35.6–55.7]	…
5–<15	348	40.5 [33.1–48.4]	.0095	26.4 [20.7–33.1]	.0093	54.3 [46.6–61.8]	.004
≤15	768	32.2 [27.7–37.0]		18.6 [15.1–22.7]	…	41.4 [36.3–46.7]	…
Sex (n = 1237)							
Male	705	36.8 [31.6–42.4]		23.3 [18.6–28.8]	…	48.1 [42.4–53.9]	…
Female	532	32.3 [27.1–38.1]	.065	20.3 [16.6–24.7]	.242	43.4 [37.7–49.3]	.095
Index/neighbor (n = 1241)							
Index	513	36.3 [30.8–42.3]		21.9 [17.2–27.3]	…	47.7 [41.3–54.0]	…
Neighbor	728	33.2 [27.6–39.3]	.529	21.3 [17.0–26.3]	.993	44.2 [37.9–50.6]	.526
Bed net use (all; n = 1241)							
No	966	32.5 [28.0–37.3]		21.1 [17.1–25.7]	…	43.8 [38.6–49.2]	…
Yes	275	40.2 [30.1–51.1]	.066	22.7 [16.2–30.9]	.729	50.6 [40.5–60.7]	.07
Bed net use (Hamsafej excluded; n = 1116)							
No	950	31.8 [27.3–36.6]		20.8 [16.9–25.4]	…	43.3 [38.1–48.7]	…
Yes	166	26.8 [19.2–36.0]	.299	20.7 [13.2–30.9]	.994	39.4 [30.3–49.3]	.739
Travel (n = 1241)							
No	989	35.4 [30.7–40.4]		20.5 [16.6–25.1]	…	45.8 [40.5–51.2]	…
Yes	252	31.0 [22.7–40.6]	.228	25.4 [18.4–34.0]	.016	44.8 [35.6–54.4]	.544
Altitude (n = 1241)							
<2000	835	40.8 [35.1–46.9]		19.6 [15.3–24.8]	…	49.3 [43.1–55.6]	…
2000–2500	273	22.7 [16.7–30.0]	<.0001	31.9 [24.2–40.7]	.0001	44.0 [34.4–54.0]	.0007
>2500	133	18.8 [11.0–30.3]		12.0 [4.9–26.6]	…	25.6 [15.3–39.6]	…

*P* values and CIs were calculated using a mixed-effects logistic model including household and cluster as random effects.

Twenty-two point two percent (275/1241) of individuals reported using a bed net. Prevalence tended to be higher among individuals using a bed net (Table 2). This effect was no longer seen when Hamsafej, the only site with a recent bed net campaign and reported bed net usage of 90.4%, was excluded (Table 2). All households in Hamsafej, but only 2 households in other kebeles, reported having received IRS recently, precluding an analysis of the impact of IRS on prevalence.

### Prevalence by Site and Across Altitudes

**Figure 2.**
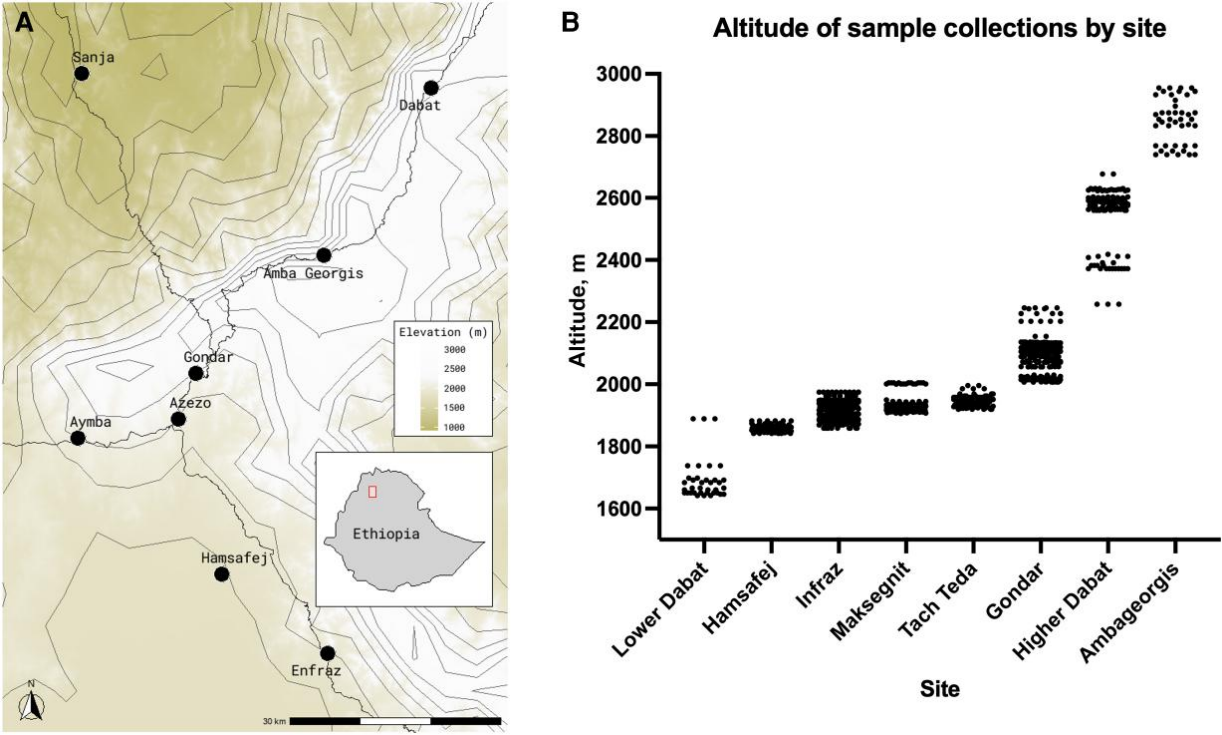
A, Locations of sample collections. B, Distribution of subclinical samples analyzed across altitudes and across sites.

Samples were collected in households from 7 kelebes, spanning an altitude range from 1642 to 2955 m (Figure 2, Table 3). In Dabat, some samples were collected at low altitudes, and others at high altitudes. These samples were analyzed separately. In 4 sites, all samples were collected within a relatively narrow altitude range of 40–116 m, while in the other 4 sites collections spanned 215–420 m (Figure 2B).

**Table 3.** Prevalence by qPCR and Proportion of Individuals Reporting Recent Travel by Site

Site Name	Altitude Range	Mean Altitude	No. of Samples Collected	% Travelers	*P. falciparum* [95% CI], %	*P*	*P. vivax* [95% CI], %	*P*	Combined [95% CI], %	*P*
Lower Dabat	1642–1899	1692	36	25.0	16.7 [13.4–20.4]		19.4 [13.3–81.2]	…	33.3 [8.8–72.1]	…
Hamsafej	1842–1882	1857	125	16.8	67.2 [51.7–79.7]		28.0 [16.8–42.7]	…	72.8 [56.4–84.7]	…
Infraz	1859–1975	1905	319	19.7	43.9 [35.6–52.5]		21.9 [15.0–30.9]	…	54.9 [46.1–63.4]	…
Maksegnit	1906–2005	1926	283	14.8	34.6 [26.9–43.3]		16.6 [9.5–27.4]	…	41.3 [31.3–52.2]	…
Tach Teda	1920–1996	1945	89	6.7	23.5 [15.6–34.2]	<.0001	11.2 [5.8–20.8]	0.0001	30.3 [20.7–42.0]	<.0001
Gondar City	2007–2246	2089	232	25.0	22.0 [16.1–29.2]		34.5 [26.0–44.1]	…	45.3 [35.1–55.8]	…
Higher Dabat	2258–2678	2545	114	28.9	18.4 [9.5–32.7]		10.5 [4.2–24.0]	…	25.4 [14.2–41.2]	…
Ambageorgis	2740–2955	2848	43	46.5	16.3 [5.0–41.8]		14.0 [1.2–67.9]	…	23.3 [6.7–56.3]	…
Total	…	…	1241	20.3	34.5		21.5	…		45.6

*P* values were calculated using a mixed-effects logistic model including household and cluster as random effects and treating site as a categorial variable.

**Figure 3.**
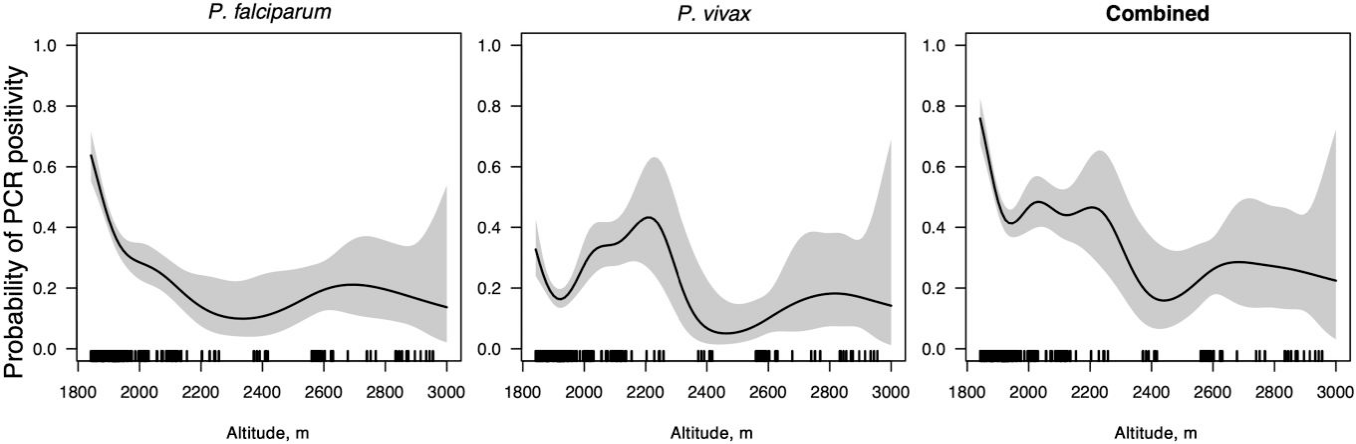
Generalized additive model of polymerase chain reaction positivity vs altitude. Shaded area shows 95% CIs. Lines along the x-axis represent altitudes of households sampled (from most households >1 sample was collected). Abbreviation: PCR, polymerase chain reaction.

*P. falciparum* prevalence by qPCR decreased with increasing altitude. Prevalence ranged from 16.3% in Ambageorgis and 18.4% in Higher Dabat (the 2 sites at the highest altitudes) to 67.2% in Hamsafej (along Lower Dabat, the site at the lowest altitude; *P* < .0001) (Table 3). The pattern for *P. vivax* was less clear; prevalence ranged from 10.5% in Higher Dabat and 11.2% in Tach Teda to 34.5% in Gondar, with lower prevalence in some sites at lower altitudes (*P* = .0001) (Table 3). Of note, *P. falciparum* prevalence was highest and *P. vivax* prevalence second highest in Hamsafej despite this being the only kebele reporting recent IRS and bed net distributions. Of 133 samples collected at altitudes >2500 m, *P. falciparum* prevalence was 18.8% (25/133) and *P. vivax* prevalence was 12.0% (16/133). In a mixed-effects logistic regression model including household and cluster as random effects, *P. falciparum* prevalence significantly decreased with altitude (19.4% decrease per 100-m increase in altitude; *P* < .001), while the effect did not reach significance for *P. vivax* (3.5% decrease per 100-m increase in altitude; *P* = .424). A generalized additive model yielded similar results (Figure 3).

### Travel as Risk Factor for Infection

Overall, 20.3% (252/1241) of individuals reported travel to a different district in the 4 weeks before sample collection. Recent travel did not affect the risk of infection with *P. falciparum* (*P* = .228) but resulted in a moderate increase in prevalence of *P. vivax* (25.4% vs 20.5%; *P* = .016) (Table 2). The proportion of individuals reporting recent travel differed significantly among sites (range, 6.7%–46.5%; *P* < .001) (Table 3), with more individuals reporting travel at higher altitudes. When each site was assessed individually, travel was not a risk factor for infection with *P. falciparum* by qPCR (*P* > .05 for all sites). For *P. vivax*, travel was not a risk factor for any site (*P* > .05) except for Gondar. There, 51.7% (30/58) of individuals reporting recent travel tested positive by qPCR, compared with 28.7% (50/174) of individuals not reporting recent travel (*P* < .001).

Subclinical infections might have been acquired many months before the survey; thus data on recent travel will not always identify imported infections. To further investigate the proportion of infections that might have been imported from the lowlands, 1775 microscopy-positive individuals were enrolled in 9 health centers at altitudes from 1000 to 2833 m, and recent travel was recorded (Table 4). The proportion of patients reporting recent travel ranged from <5% in 4 health centers at altitudes <2000 m to 21.4% at Azezo Health Center, which is at 2611 m, and 100% at Ambageorgis Health Center, which is located at 2955 m. Men reported travel significantly more often than women (19.2% vs 5.0%; *P* < .001). The proportion of *P. falciparum*, *P. vivax*, and mixed clinical infections differed significantly between health centers (*P* > .001) (Table 4).

**Table 4.** Species Composition by Microscopy and Proportion Reporting Recent Travel Among Microscopy-Positive Patients in Health Centers

		No.	*P. falciparum*, %	*P. vivax*, %	Mixed, %	*P*	% Reporting Recent Travel	*P*
Sex								
Male		1119	40.2	52.0	7.8		19.2	…
Female		644	38.5	55.9	5.6	.119	5.0	<.001
Travel								
No		1526	38.5	54.5	6.9		…	…
Yes		249	45.8	47.4	6.8	.087	…	…
Health center	Altitude	…	…	…		…	…	…
Sanja/Mussie Bamb	1000	323	46.1	44.9	9.0	…	16.1	…
Hamsafej	1860	256	23.0	59.4	17.6	…	3.5	…
Aymba	1900	167	57.5	32.3	10.2	…	1.8	…
Teda	1900	335	34.6	62.4	3.0	<.001	4.8	<.001
Enfraze	1934	164	52.4	45.7	1.8	…	3.0	…
Azezo	2150	333	41.4	56.2	2.4	…	13.2	…
Dabat	2611	98	25.5	74.5	0.0	…	21.4	…
Ambageorgis	2833	99	33.3	55.6	11.1	…	100.0	…

*P* values show differences in species composition or proportion reporting recent travel calculated by Chi-square test.

### *P. falciparum hrp2*/*3* Deletion Status and Ability of HRP2 vs LDH-Based RDTs to Diagnose Subclinical Infections

The HRP2-based RDT used for parasite screening was the product in use in Ethiopia at the time of the study. Two hundred eighteen *P. falciparum*–positive subclinical infections were typed for *hrp2*/*3* deletion; 98.2% (214/218) carried *hrp3* deletion, and 46.3% (101/218) *hrp2* deletion.

To determine whether LDH-based RDTs would detect more infections, 361 samples were collected from subclinical individuals. By qPCR, *P. falciparum* prevalence was 28.5% (103/361) and *P. vivax* prevalence was 28.8% (104/361). No differences in prevalence were observed between age groups <5, 5–15, and >15 years (*P* = .633 for both species combined), or between males and females (*P* = .315). The HRP2 target on the Biocredit was more sensitive than the HRP2 target on the Bioline Malaria Ag Pf/Pv (McNemar *P* = .0013) and CareStart (McNemar *P* = .0027) tests. On the Biocredit RDT, among 103 samples confirmed *P. falciparum* positive by qPCR, 35 samples were detected by both targets, 28 by LDH only, and 2 by HRP2 only (McNemar *P* < .001). Of 21 samples positive for LDH only that were typed for *hrp2*/*3* deletions, all except 1 carried double deletions. The Bioline and Carestart RDTs detected 27%–28% of *P. vivax* infections, and microscopy detected 11.5% (Table 5).

**Table 5.** Sensitivity, Specificity, PPV, and NPV of Different RDTs and Microscopy Compared With qPCR Sensitivity Data Are Given for All Samples Positive by qPCR and for Samples at Densities >20 Parasites/µL by qPCR

	Sensitivity All Densities [95% CI], %	Sensitivity >20 Parasites/µL [95% CI], %	Specificity, %	PPV, %	NPV, %
*P. falciparum*					
Abbott	30.1 (31/103) [21.5–39.9]	44.4 (20/45) [29.6–60.0]	99.2 (256/258)	93.9	78.0
Combo	31.1 (32/103) [22.3–40.9]	46.7 (21/45) [31.7–62.1]	99.6 (257/258)	97.0	78.4
Bioredit HRP2	35.9 (37/103) [26.7–46.0]	51.1 (23/45) [35.8–66.3]	97.3 (251/258)	84.1	79.2
Bioredit LDH	61.2 (63/103) [51.1–70.6]	91.1 (41/45) [78.8–97.5]	97.3 (251/258)	90.0	86.3
Biocredit combined	63.1 (65/103) [53.0–72.4]	93.3 (42/45) [81.7–98.6]	95.7 (247/258)	85.5	86.1
Microscopy	31.1 (32/103) [22.3–40.9]	51.5 (23/45) [35.8–66.3]	97.3 (251/258)	82.1	78.0
*P. vivax*					
Abbott	27.9 (29/104) [19.5–37.6]	37.2 (29/78) [26.5–48.9]	96.9 (149/257)	78.4	66.5
Combo	26.9 (28/104) [18.7–36.5]	35.9 (28/78) [25.3–47.6]	95.7 (246/257)	71.8	76.4
Microscopy	11.5 (12/104) [6.1–19.3]	15.4 (12/78) [8.2–25.3]	98.1 (252/257)	70.6	73.3

Specificity for *P. falciparum* was >99% for the Bioline and CareStart RDTs and 97.3% for each line of the Biocredit RDT. Among samples that were false-positive by the Biocredit RDT, 3/11 were positive for both bands, and 4 each for HRP2 or LDH only. Of 12 samples false-positive by microscopy (7 *P. falciparum*, 5 *P. vivax*), 10 were positive for the other species by qPCR, and only 2 were negative for either species.

## DISCUSSION

A high prevalence of subclinical *P. falciparum* and *P. vivax* infection was found at high altitudes, even above 2500 m. The vast majority of infected individuals did not report recent travel. The same was the case for confirmed clinical cases, except for one health center located >2800 m. The data are in line with previous studies conducted in the highland fringe zone and other sites in the East African highlands that found that many clinical cases did not report recent travel [13, 20]. A recent *P. falciparum* genotyping study found clonal and closely related genotypes among clinical infections in Gondar [21]. This suggests local transmission, as it would be improbable that many imported infections share the same genotype. A decline in *P. falciparum* prevalence with increasing altitude was observed, but no clear trend was found for *P. vivax*. *P. vivax* is known to be able to develop in the mosquito at lower temperatures than *P. falciparum*, contributing to its geographically wider distribution [22].

Several limitations might have resulted in imprecise estimates of prevalence and in an underestimate of the proportion of imported infections. Many subclinical infections were likely acquired many months before the survey; thus data on recent travel would fail to identify them as imported. Self-reported travel histories of clinical cases at health centers, however, confirmed that many patients had not traveled, except for the highest health center. Travel data might also have missed short-term trips to lower altitudes within the same district. *P. vivax* relapses cannot be distinguished from primary infections. Both subclinical and clinical *P. vivax* infections might be the result of relapses, and thus not the result of recent mosquito bites. In the case that subclinical infections cluster around clinical infections, the inability to collect samples from control households would have resulted in an overestimation of prevalence [23, 24]. Yet, prevalence in samples collected in the highland fringe zone for the comparison of RDTs (where no follow-up of clinical cases was done) showed virtually identical prevalence. Further studies including blood sample and mosquito collections will be needed to determine the intensity of local transmission at high altitudes.

As a result of the general assumption that malaria is rare in the Ethiopian highlands, coverage of malaria control interventions was poor. Excluding Hamsafej, where a bed net campaign and IRS had taken place before sample collection, less than a fifth of individuals reported using a bed net, and no recent IRS had been conducted. In the highland fringe zone at 1750–2000 m, until 2019, all kebeles in Gondar Zuria district received regular IRS and bed nets. Scheduled IRS campaigns in September 2020 were canceled due to COVID-19 [25]. The September IRS used to suppress the mosquito population before the main rainfalls from October to December [25]. In 2022, control resumed but only in 8 kebeles, including Hamsafej. Despite high coverage of IRS and bed nets, which were found to reduce prevalence in other countries [26], prevalence in Hamsafej remained high. The data corroborated personal communications from NMCP and health center staff who reported that the impact of IRS was not good, with mosquitos surviving on sprayed walls and increasing case numbers.

In elimination and pre-elimination settings, the WHO conditionally recommends reactive case detection (RCD) to shrink the subclinical reservoir [27]. Other strategies such as mass or focal test and treat might also be considered [28]. RDTs are typically used for such programs. In countries where *hrp2* deletions are reported, the WHO recommends switching to alternative diagnostic tools if *hrp2*/*3* deletions result in false-negative diagnosis in >5% of clinical cases [29]. At this frequency of deletion, it is expected that more infections are missed because of deletions than would be missed because alternative diagnostics might be less sensitive. No guidelines and limited data exist to determine what RDTs should be used to screen for subclinical infections in sites with *hrp2* deletions. Given the lower density of subclinical infections, HRP2/3-based RDTs might be used even if they miss >5% of infections, as alternative diagnostics might miss even more. In the current study, the LDH-based RDT detected >60% of PCR-positive infections, nearly twice as many as HRP2-based RDTs. LDH-based RDTs thus are recommended for population screening for subclinical infections. While a substantial number of PCR-positive infections were missed by either RDT target, they were very low density and less likely to be infectious to mosquitos [7, 30]. The proportion of *P. vivax* infections detected by RDT was low. More sensitive diagnostic methods will be needed for RCD or focal test-and-treat campaigns to be successful in reducing *P. vivax* transmission. Antibody-based screening for hypnozoites and, following testing for G6PD deficiency [31], primaquine or tafenoquine administration to reduce *P. vivax* relapses might be required [32, 33].

In conclusion, prevalence levels in the Ethiopian highlands are high, and many individuals presenting with clinical malaria did not report recent travel. Intensified malaria control action is needed in order to bring the area back to pre-elimination status.

## Supplementary Material

ofag247_Supplementary_Data

## Supplementary Data

Supplementary materials are available at *Open Forum Infectious Diseases* online. Consisting of data provided by the authors to benefit the reader, the posted materials are not copyedited and are the sole responsibility of the authors, so questions or comments should be addressed to the corresponding author.
